# Neurocognitive Dysfunctions and Their Therapeutic Modulation in Patients With Methamphetamine Dependence: A Pilot Study

**DOI:** 10.3389/fpsyt.2020.00581

**Published:** 2020-07-02

**Authors:** Nadine Bernhardt, Johannes Petzold, Cornelius Groß, Anna Scheck, Shakoor Pooseh, René Mayer-Pelinski, Ulrich S. Zimmermann, Michael N. Smolka, Maximilian Pilhatsch

**Affiliations:** ^1^ Department of Psychiatry and Psychotherapy, Technische Universität Dresden, Dresden, Germany; ^2^ Freiburg Center for Data Analysis and Modeling, Albert-Ludwigs-Universität Freiburg, Freiburg, Germany; ^3^ Department of Addiction Medicine and Psychotherapy, Isar-Amper-Klinikum München-Ost, Haar, Germany; ^4^ Department of Psychiatry and Psychotherapy, Elblandklinikum Radebeul, Radebeul, Germany

**Keywords:** methamphetamine, crystal meth, sustained attention, delay discounting, psychotherapy

## Abstract

**Aim:**

Methamphetamine (MA) abuse and dependence are increasing worldwide and are commonly associated with cognitive deficits. Some studies indicate that such impairments can improve if users become abstinent, but overall results remain inconclusive. Hence, we have performed a longitudinal case-control study investigating key surrogates for attention and impulsive decision-making before and after treatment.

**Methods:**

Thirty patients with MA dependence and 24 non–substance-abusing control participants were recruited. Groups were matched on age, sex and education. All subjects performed a baseline assessment to obtain neurocognitive measures of sustained attention and delay discounting. Patients subsequently participated in an MA-specific relapse prevention program including repeated monitoring of relapse status. After 3 months, participants of both groups were reevaluated for neurocognitive performance.

**Results:**

At baseline, MA patients showed a significantly higher number of omissions compared to controls, indicative of lower sustained attention. Interestingly, we observed a steep decrease of omissions in MA patients to control-group level post treatment. On the other hand, MA patients discounted delayed rewards significantly stronger than controls, indicating a more impulsive choice behavior both before and after treatment.

**Limitation:**

The results should be interpreted with care because of the small sample and short follow-up period.

**Conclusion:**

Our data support earlier findings on partial recovery of cognitive deficits in MA patients. They also strengthen the indication for recently recommended psychotherapeutic interventions and may provide a behavioral monitoring tool to inform treatment progress.

## Introduction

Methamphetamine (MA) also called “crystal meth” is a psychostimulant, whose use has become increasingly popular in several European countries (1). This development reflects its comparably low costs and highly addictive properties. MA abuse and dependence are associated with numerous adverse consequences, which are of great public concern. For example, MA users are more likely unemployed and experience a number of interpersonal difficulties (2). Furthermore, MA users are at high risk for mental and physical health conditions, including depression, anxiety, psychosis, suicide, sexually transmitted diseases and cardiovascular complications (3–5). Consequently, doctors and staff in hospitals, private practices and addiction treatment centers encounter increasing numbers of subjects who suffer from severe complications of MA use. The growing prevalence of MA dependence in Germany prompted the federal government to initiate the development of MA-specific treatment guidelines (6). The areas that are most affected include parts of Saxony, Thuringia and Bavaria. Pharmacotherapy has shown limited effectiveness, making psychotherapeutic interventions the treatment of choice (7). These include cognitive behavioral therapy, motivational interviewing and contingency management, which aim to reduce drug use, positive urine samples and craving. However, such recommendations vastly rely on the transfer of knowledge from overseas and may not be representative of the specific characteristics that are experienced locally. This is especially relevant when mechanistic aspects are not yet fully clarified, which include above all cognitive dysfunctions in MA users and their course under therapy. As such more research is urgently needed to strengthen the evidence for the recommended psychotherapeutic interventions and optimization of care (8).

Compared with other stimulants, MA has a more lipophilic structure and a very long half-life of 8–13h, causing a fast onset and long duration of action in the brain (9). Besides the resulting highly addictive potential through the acute modulation of the monoaminergic system (10), long-term MA exposure produces persistent damage to dopamine and serotonin release in nerve terminals and triggers gliosis and apoptosis (11). Moreover, chronic MA abuse is associated with abnormalities in brain structure, metabolism and functions, predominantly within the frontostriatal and limbic systems (12). Such changes reflect cognitive impairments (13, 14) with pronounced alterations in multiple aspects of attentional control, working memory and executive functions including decision-making (15–19). Clinically, MA-dependent individuals appear distractible and exhibit difficulties in sustaining attention. The ability to keep one’s mind continuously focused is considered a fundamental dimension of attentional control with relevance to higher cognitive processes (20). Chronic MA abusers generally show poorer performance than controls on several attention tasks [(18), e.g. CPT and Stroop tasks (19, 21, 22)], linked to MA-associated neuronal damage and network activity in the cingulate and insular cortices (23). Another cognitive domain altered in MA-dependent individuals is impulsive choice behavior with higher rates of delay discounting relative to controls, i.e., the propensity to select an immediate reward at the expense of a greater future reward (24–28). Overly steep discounting is consistently correlated with a range of conditions, including various drug addictions, obesity and schizophrenia (29–32), and suggested to play a causal role in upholding maladaptive behaviors (continued drug taking despite positive long-term outcome of abstinence, e.g., treatment, health, social). Delay discounting in MA abuse is associated with prefrontal inefficiency, an indication of more automatic and diminished deliberate decision-making processes (e.g. habitual response to a cue signaling drug availability) (24). Such impairments in attention and decision-making may thus critically undermine the individual’s efforts to stop or reduce MA use, thereby negatively affecting the outcome of treatment including cognitive behavioral strategies. Indeed, a higher number of omissions of target stimuli in attention tasks has been found to predict relapse among recently abstinent MA-dependent patients (33, 34). While maladaptive decision-making has been shown to predict dropout (34), altered neural activity during decision-making may predict relapse (35).

Despite considerable research on adverse functional consequences of chronic MA use and their importance for successful long-term treatment outcomes, the extent to which these problems persist following periods of abstinence remains controversial. Impairments associated with MA use tend to improve with increasing abstinence duration (36–38). The amelioration of cognitive deficits has been shown for short intervals of several weeks (39–41) including attention (42), while other studies have demonstrated that the reinstatement of especially dopamine signaling and associated cognitive functioning may take months to years (16, 36, 43, 44). Moreover, it is still debated whether some of the MA-induced cognitive deficits may be irreversible [e.g. (45)].

The aim of this study was to examine sustained attention and impulsive decision-making in MA-dependent patients and the changes in these domains following a new standardized psychotherapeutic intervention. In addition, we included a healthy comparison sample to help distinguish actual improvement in neuropsychological functioning over time from practice effects. Consistent with previous evidence for partial neurobiological, neuropsychological and psychiatric recovery following treatment of MA-dependent individuals, we hypothesized that sustained attention would improve over a 3-month period while more temporally stable individual characteristics of impulsive choice (46–48) would remain unaltered.

## Methods

### Participants

**Figure 1:**
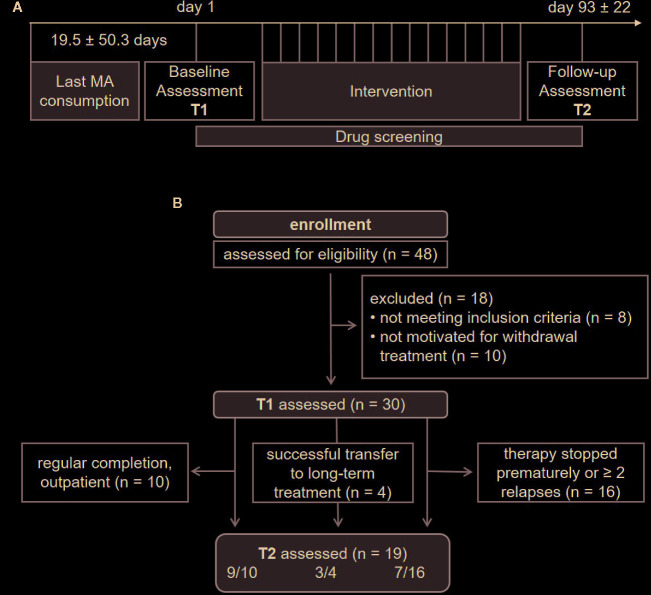
Study overview, recruitment details and sample size of methamphetamine (MA) patients throughout the study process. T1, baseline assessment; T2, follow-up assessment.

Patients were recruited at the University Hospital Dresden. Study inclusion criteria for MA patients were 18–65 years of age; meeting the diagnostic criteria for MA abuse or dependence according to the International Classification of Diseases (ICD-10); abstinence from illicit drug use for at least 2 days, proven with negative urine screening results for MA, amphetamines, MDMA, opioids, and THC. Exclusion criteria were any medical conditions, e.g. schizophrenia, severe depressive episodes or limited physical mobility, interfering with the capability to attend group therapy. The assessment of psychiatric comorbidities was supported by a standardized interview using the M.I.N.I. International Neuropsychiatric Interview (49). In addition, non–substance-abusing control subjects (HC) matched for age, sex, and education were recruited *via* advertisements placed on local community-based websites, which offered employment and volunteer opportunities. HC participants were required to have no lifetime experience with any kind of stimulant (MA, amphetamines, MDMA, methylphenidate, cocaine, etc.) and to have never been diagnosed with drug addiction or suspected of having a substance use disorder. Moreover, the presence or history of any psychiatric disease was excluded by applying a standardized questionnaire (including questions such as, “Have you ever been diagnosed with any mental illness?”). In cases of doubt, a psychiatrist was consulted. The final sample consisted of 30 MA-dependent patients (**Figure 1**) and 24 HC subjects. All participants provided written informed consent and received a compensation between 50 and 90€. The study was approved by the local ethics committee of the Technische Universität Dresden and carried out in accordance with the Declaration of Helsinki.

### Study Design

Research staff independent of the relapse prevention program conducted the study recruitment as well as baseline (T1) and follow-up (T2) assessments. Assessments comprised a standardized interview to collect socio-demographic information such as age, sex, partnership, migration status, number of children, school, and vocational qualifications as well as current employment status. Participants of both groups then completed a neuropsychological assessment, which encompassed sustained attention and delay discounting as key surrogates for executive function and impulsive decision-making, respectively. After 3 months, subjects completed a follow-up with the same neuropsychological assessment (**Figure 1A**).

### Treatment

In- and outpatients were enrolled in our MA-specific relapse prevention program, which is an adaption of the manual by (50) and has demonstrated good feasibility (51) and effectiveness (52) in daily clinical routine. Up to six participants attended 15 twice-a-week group sessions, which lasted 50 min. Sessions were conducted by a psychologist, and the treatment method was based on motivational interviewing. The program’s progress and goals emphasized on determining high-risk situations for MA use, providing skills to resolve personal, social and environmental barriers, and enhancing coping methods to prevent relapse. Before the first module, one individual session took place in which information about the group program and rules was provided, and the willingness to participate was discussed. In detail, the following topics were covered: “Explanation of the Therapeutic Rational” (module 1), “Motivational Clarification” (modules 2 and 6, if necessary repeated throughout therapy), “Craving” (3–5; psychoeducation, identification of triggers, strategies), “Social Risk Situations” (7–9; dealing with social risk situations, rejection training in role plays), “Apparently Harmless Decisions” (10 and 11, psychoeducation and dealing with seemingly harmless decisions), “Personal Risk Situations” (12 and 13, dealing with personal risk situations), “Emergency Plan” (14, preparation of an emergency plan with strategies for coping with high-risk situations), “Change Plan” (15, coping with problems after the end of therapy).

Inpatients provided weekly urine samples. Outpatients were also called unannounced, but randomized in the morning between Monday and Friday with a probability of 1/6 and ordered to provide urine for drug screening. Urine sample delivery took place under personal observation. In addition, manipulations were made more difficult by measuring the urine temperature immediately after delivery. Samples were quantitatively analyzed at the Institute of Legal Medicine at the Technische Universität Dresden and a sum of 300 ng/ml for MA and amphetamines was set as a cut-off for a positive result. Relapse was defined as any positive urine result during the course of treatment.

Treatment was classified as “successful” if the patient was enrolled in a postacute management program or attended at least 8 of 15 group therapy sessions. In addition, maximum one MA relapse until T2 indicated by negative drug tests was allowed, provided the relapse was admitted and self-critically processed. If the therapy was prematurely terminated without following specific long-term treatment or if two relapses occurred, treatment was classified as “unsuccessful”. “Retention rate” was assessed as the quotient between number of successful treatment and total number of patients completing T1.

### Tasks and Measures

#### Clinical Data

At T1, MA usage patterns were assessed, which included age of first MA use, total duration of MA use and days of abstinence. Psychiatric comorbidities were recorded according to ICD-10 criteria. A positive family history for the presence of mental disorders in 1^st^ and 2^nd^ degree relatives was systematically inquired according to (53).

#### Sustained Attention

The sustained attention subtest (SA) of the reliable and valid computer-administered test of Attentional Performance 2.3.1 (54) was used, which takes approximately 15 min. Participants were rapidly presented with individual symbols varying in shape, size, and filling (e.g. a small triangle and a large circle) and asked to press a key if a symbol matched the shape of the symbol presented immediately before. Omissions were recorded as dependent measures, with higher scores reflecting poorer sustained attention (34). Further variables recorded included the number of incorrect answers (errors) and response times.

#### Impulsive Choice

We used a delay discounting task (DD) in which participants repeatedly needed to choose a smaller immediate amount of money or a greater delayed one (e.g. 2€ now or 8€ in 1 week). Offers were randomly displayed on the left or right side of the screen. There was no time limit for making decisions. To ensure realistic choices and increase task relevance, subjects were informed that at the end of the experiment, one trial would be selected randomly and paid according to the given choice. Monetary rewards ranged from 0.30 to 10€. The subjective evaluation of the offers has been described by a hyperbolic function [e.g. (55)]: V = A/(1 + *k*d) , where V represents the subjective value of the amount A after a delay d (3, 7, 14, 31, 61, 180, or 365 days) and *k* is a free parameter representing the discount rate. Larger *k* values represent preference for immediate amounts, which has been interpreted as impulsive choice behavior. To provide behavioral estimates, an adaptive procedure for binary choice presentation was used. For a detailed description of the mathematical framework see (56) and for an application of the task in a clinical cohort of patients with alcohol use disorder see (32). Briefly, a trial-by-trial adaptive approach was chosen to present participants with choice options near their individual indifference point at each trial, thus allowing for fast assessment of individual parameters of behavior. The likelihood of choosing between the two offers followed a softmax probability function: *P* (*a*1| *k*, β) = 1/(1 + exp [β (V2 - V1)]), where V1 and V2 are the subjective values of the offers and *β* > 0 serves as a consistency parameter. The algorithm started from liberal prior distributions on the parameters and, after observing a choice at each trial, updates the belief about the parameters using the Bayes’ rule: *P* (*k*, β|*choice*) ∝ *P* (*k*, β|*choice*) *P* (k, β). The procedure continued for 50 trials and the estimates at the final trial were considered the best-fitting parameters for a participant. The distribution of parameter estimates over task progression was plotted and found to converge well, yielding stable final estimates of choice behavior (**Supplementary Figure 1**). Recorded variables included: log[*k*] as an estimate of discounting behavior, log[*β*] as a measure of consistency of choice behavior and for each trial the time to make a decision.

### Data Analysis

Data were initially analyzed with SPSS version 25. Histograms, box plots, and Shapiro tests were employed to judge parameter distribution. Differences between groups in socio-demographic and cognitive variables were evaluated using appropriate parametric or nonparametric tests comparing two independent groups as specified in **Tables 1** and **2**. Logistic regression analyses with forward stepwise selection was used for outcome prediction in MA patients as previously described (52). Individual median response times over all trials were used for group-wise analysis. Longitudinal analyses of pre- and posttreatment cognitive assessments were conducted using R 3.2.3 (R Development Core Team, 2015). We used mixed-effects models (lmer, R-package: lme4); for DD: prediction of log[k] out of time, group and their interaction; for SA: prediction of omissions out of time, group and their interactions. Following, we assessed the effect of comorbidity on the results found using mixed-effects models including the factors time and the presence/absence of an additional psychiatric disorder. We report estimates, standard deviations, t‐values and p-values derived using Satterthwaite approximations. An alpha level of 0.05 was set for the determination of statistical significance.

**Table 1:** Socio-demographic and clinical data at T1 and T2.

Characteristic	Group		Comparison	
	MA (n = 30)	HC (n = 24)	*Test value*	*p*
***Socio-demographic data***				
Age (years)	29.0 ± 6.8	28.8 ± 5.6	0.121^a^	.904
Sex (females)	36.7 (11)	33.3 (8)	0.065^b^	.799
Partnership	43.3 (13)	62.5 (15)	1.962^b^	.161
Migration	2.5 (1)	7.5 (3)	1.634^b^	.201
Children	63.3 (19)	50.0 (12)	−1.335^c^	.182
Education*	76.7 (23)	70.8 (17)	0.236^b^	.627
Unemployment	70.0 (21)	20.8 (5)	13.900^b^	**.001**
***Clinical data***				
Age of first MA use (years)	19.2 ± 5.2	N/A	N/A	N/A
MA abuse duration (years)	7.0 ± 4.3	N/A	N/A	N/A
Abstinence (days)	19.5 ± 50.3	N/A	N/A	N/A
Psychiatric comorbidities	53.3 (16)	N/A	N/A	N/A
Medication	16.7 (5)	N/A	N/A	N/A
FH+	43.3 (13)	N/A	N/A	N/A

Descriptive statistics (mean ± SD or % (N)) and results of group differences. MA, methamphetamine patients; HC, healthy controls; FH+, positive family history for psychiatric disorders according to first-degree relatives.

*secondary school or lower.

a t (paired t-test).

b Pearson chi-square (exact chi-square test).

c Z (Wilcoxon matched pairs rank sum test). In bold: significant at p < 0.05.

**Table 2:** Summary statistics of experimental parameters.

	T1					
	MA (n = 30)	HC (n = 24)	*Test value^a^*	*p*		*d*
***Sustained Attention***						
errors	8.93 ± 19.10	10.79 ± 37.02	-0.238	.812		−0.07
omissions	10.63 ± 8.48	4.00 ± 3.65	3.569	**.001**		0.98
response time (ms)	558 ± 130	515 ± 118	1.252	.216		0.34
***Delay Discounting***						
log[k]	−2.2 ± 2.3	−3.9 ± 2.1	2.565	**.013**		0.70
log[β]	−1.6 ± 1.8	−1.9 ± 1.5	0.808	.423		0.22
deliberation time (s)	2.2 ± 0.8	2.0 ± 0.6	0.935	.354		0.26
	**T2**					
	**MA (n = 19)**	**HC (n = 17)**	***Test value^a^***	***p***		
***Sustained Attention***						
errors	4.77 ± 5.31	5.61 ± 14.32	−0.231	.818		−0.08
omissions	5.44 ± 7.16	4.11 ± 4.93	−0.650	.520		0.22
response time (ms)	504 ± 107	535 ± 124	−0.796	.434		−0.26
***Delay Discounting***						
log[k]	−2.3 ± 2.4	−4.1 ± 1.7	2.451	**.021**		0.86
log[β]	−1.8 ± 1.1	−1.7 ± 1.0	−0.451	.655		−0.15
deliberation time (s)	1.8 ± 0.6	1.9 ± 0.5	−1.011	.322		−0.34

MA, methamphetamine patients; HC, healthy controls; d, Cohen’s d; log[k], discounting parameter; log[β], consistency parameter.

a t (paired t-test). In bold: significant at p < 0.05.

## Results

### Sample Description

Socio-demographic and clinical characteristics of patients and controls are summarized in **Table 1**. Statistical analyses showed no significant difference in age, sex and education. However, unemployment was significantly more frequent in MA patients as expected. Fifty-three percent (n = 16) of participants with MA dependence did present psychiatric comorbidities at the time of treatment: five suffered from unipolar depression, two from attention deficit hyperactivity disorder (ADHD), three had drug-induced psychosis before the start of the study, and one patient had posttraumatic stress disorder and dissocial personality disorder. One patient had ADHD combined with a borderline personality disorder. One patient was diagnosed with three comorbidities: ADHD, a unipolar moderate depressive episode and a borderline personality disorder. Of these 16 comorbid patients, five additionally showed a harmful use of cannabinoids and four an alcohol dependence. Five (16.7%) patients were prescribed regular psychotropic medication during the study period: one patient received doxepin, one patient sertraline, one patient olanzapine, one patient a combination treatment of duloxetine and quetiapine, and one patient methylphenidate. Clinically, none of these patients were significantly affected by the medication.

### Outcome at Follow-Up

T2 data were obtained from 70% (n = 17) of HC and 63.3% (n = 19) of MA patients initially included. Treatment outcome and participant characteristics of the extended MA patients sample (successful vs. unsuccessful) are reported elsewhere (52). Measures in our subsample (one patient diagnosed with schizophrenia excluded) were comparable. In summary, the treatment was classified as successful in 14 of 30 patients (46.6%). Four of these patients were transferred to a specific long-term treatment and 10 patients into a specific postacute outpatient treatment setting at our department. By contrast, the treatment was considered not successful in 16 cases, i.e., patients had more than one relapse with MA during the study or prematurely terminated the program (**Figure 1B**). Correlational analysis showed trend-level significance for longer regular MA use in men across groups (r = 0.359, p =.051). Moreover, patients with an unsuccessful outcome were predominantly male (81.3%). The abstinence period before baseline (T1) tended to be longer in patients with a favorable outcome (U = 71.500, z = −1.693, p =.093), without being significantly correlated with sex (ρ = −0.100, p =.597) or the duration of regular MA use (ρ = −0.134, p =.479) across groups. Among the demographic and clinical variables, the only predictor significantly increasing the odds of a successful outcome was a shorter period of regular MA use (OR = 1.342, CI 95% for OR = 1.028–1.753, b = 0.294, SE = 0.136, p =.031).

### Sustained Attention

**Figure 2:**
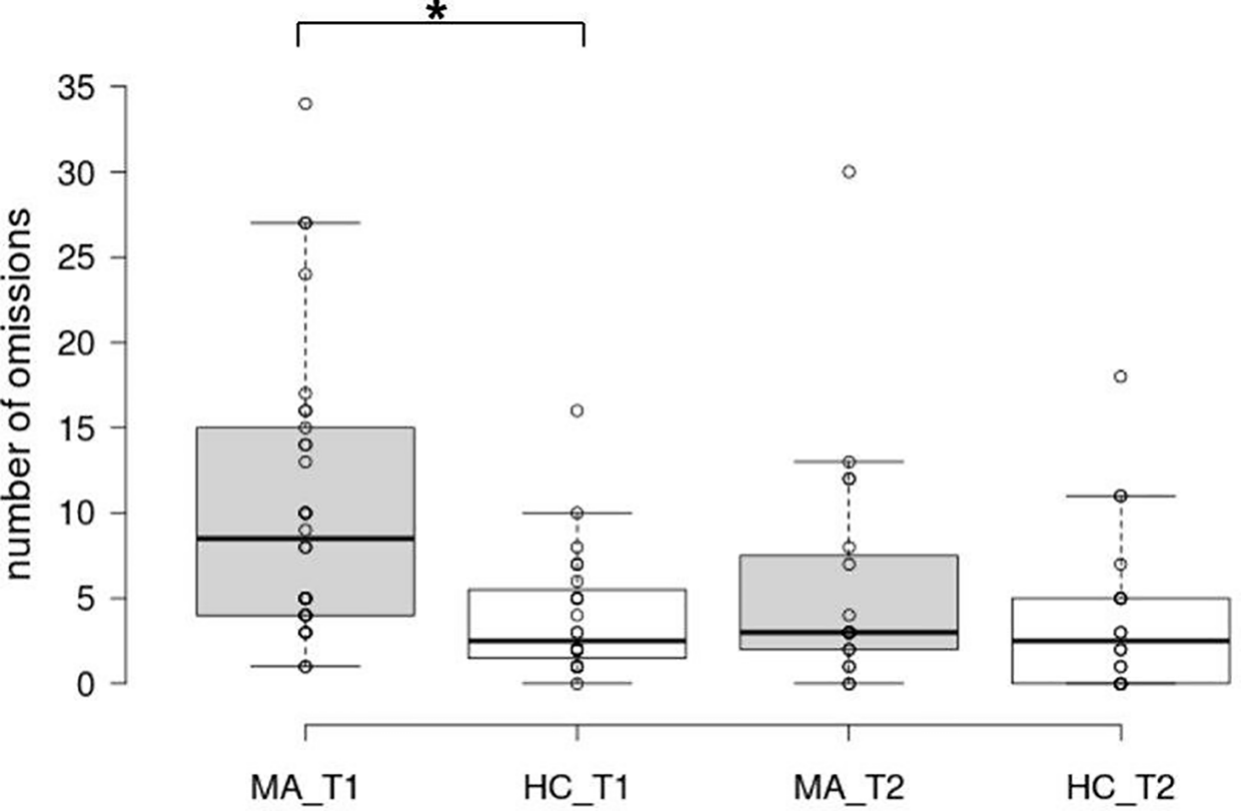
Box plots of omissions in the sustained attention task. The horizontal line represents the median; the boxes extend to the 1^st^ and 3^rd^ quartile, while whiskers extend to the max/min or the corresponding quartile + 1.5 IQR. Additionally, each data point is visualized. MA_T1, MA patients at baseline; HC_T1, control group at baseline; MA_T2, MA patients at follow-up; HC_T2, control group at follow-up; *, significant main effect of group.

Recorded variables and test statistics can be found in **Table 2**. There were no differences between groups in the number of incorrect answers (errors) and response times. Analysis of omissions over groups and time points showed a significant effect of time (Estimate = −4.66, SD = 1.45, t = −3.22, p =.003), group (Estimate = −11.3, SD = 3.31, t = −3.42, p =.001) and a significant interaction effect (Estimate = 4.66, SD = 2.09, t = 2.23, p =.032). At baseline, MA patients had significantly more omissions, indicative of poorer SA. Over time, the patient group showed a steep decline of omissions, while the control group remained on the same level (**Figure 2**). Analysis of MA patients controlling for comorbidity similarly showed a significant effect of time (Estimate = −4.35, SD = 1.71, t = −2.536, p =.002) but no effect of comorbidity (Estimate = 2.49, SD = 2.79, t = 0.892, p =.379) (**Supplementary Fig. 2**). Baseline performance in SA did not significantly differ between patients who finished the program and patients who prematurely stopped treatment (**Supplementary Fig. 3**).

### Impulsive Choice

**Figure 3:**
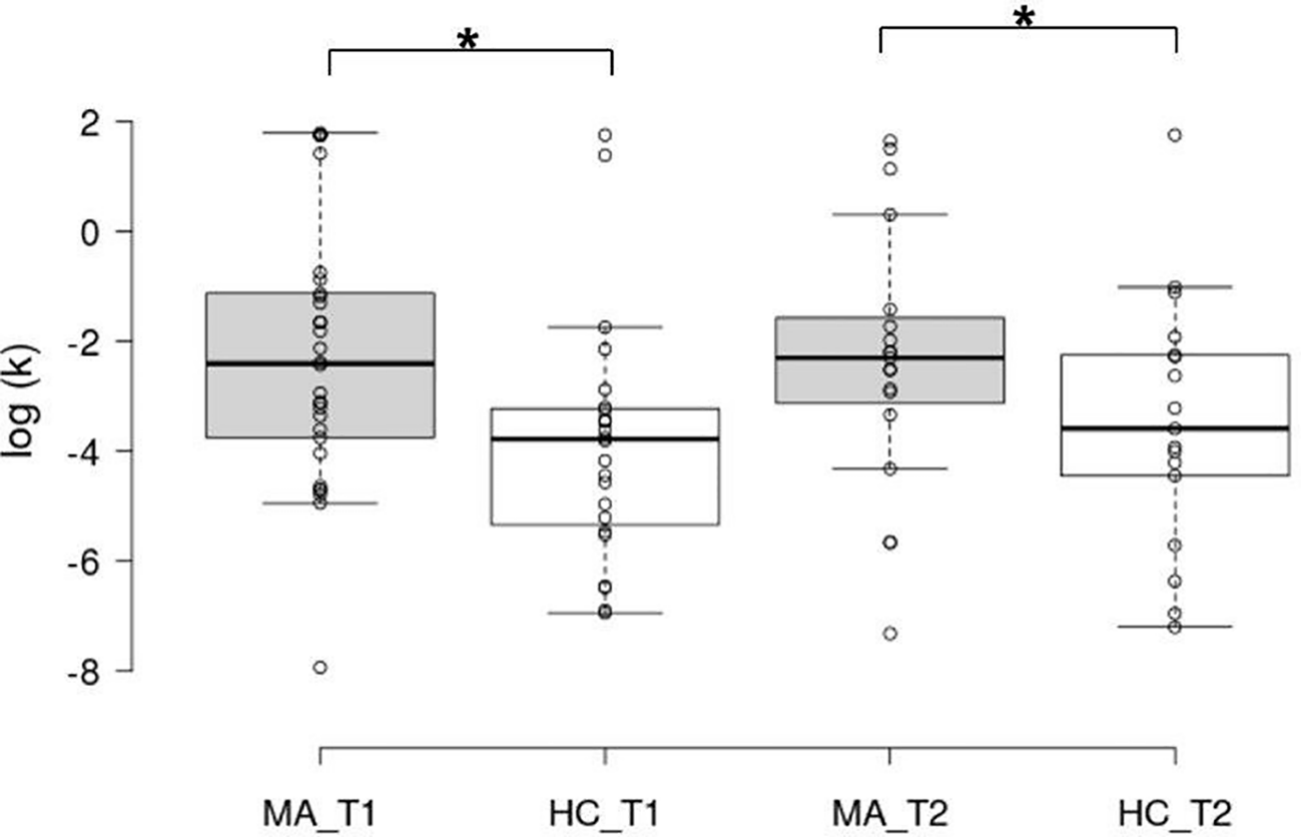
Box plots of the decision-making parameter from the delay discounting task in log scale. The horizontal line represents the median; the boxes extend to the 1^st^ and 3^rd^ quartile, while whiskers extend to the max/min or the corresponding quartile + 1.5 IQR. Additionally, each data point is visualized. MA_T1, MA patients at baseline; HC_T1, control group at baseline; MA_T2, MA patients at follow-up; HC_T2, control group at follow-up; *, significant main effect of group.

Estimates of choice behavior and deliberation times as well as test statistics can be found in **Table 2**. Analysis showed a group effect for DD (Estimate = −2.02, SD = 0.99, t = −2.04, p = 0.04) with MA patients having significantly higher estimates, indicating that they chose the immediate option more often and thus were more impulsive (**Figure 3**). For discounting estimates, there was neither a significant change in time from T1 to T2 (Estimate = 0.11, SD = 0.41, t = 0.26, p = 0.791) nor a significant interaction effect (Estimate = 0.42, SD = 0.59, t = 0.71, p = 0.487). Analysis of MA patients controlling for comorbidity similarly showed no significant effect of time (Estimate = 0.02, SD = 0.49, t = 0.04, p = 0.973) and comorbidity (Estimate = 0.59, SD = 1.44, t = 0.415, p =.681). No significant differences between groups were observed for consistency of choices and deliberation times.

## Discussion

The main aim of the present study was to determine whether cognitive impairments in attention and impulsive choice behavior in recently detoxified MA patients recover during a 3-month program, which included psychotherapy and regular drug screening. Our results showed that baseline performances in sustained attention, which were inferior compared with those of controls, improved so much during this period that they were no longer impaired at the follow-up session. In contrast, more impulsive delay discounting in MA patients compared to controls did not change over time.

Baseline differences between groups in both cognitive domains tested in this study are in line with a range of prior studies (13, 22, 24, 26, 28, 33, 43, 57–59). Thus, attentional deficits and choice behavior favoring immediate rewards are consistently associated with MA use. On the other hand, decision speed of MA patients has been found unaltered previously (25, 60) as well as in our sample in which performance demonstrated no group effect. Observed heightened impulsive behaviors may predict drug use or can be a consequence of repeated drug exposure and withdrawal (61). However, impulsivity is a multifaceted construct and impulsive choice behavior might undergo a developmental change that parallels drug consumption as directly observed in rodent studies [e.g. (62)] and suggested from human work in addiction [c.f. (32)]. On the neurobiological level, neurotoxic effects of MA and adaptive changes in the structure of brain regions involved in motivation, reward and the top-down control of behavior may be causal (21, 23, 27, 63–65). This is complemented by functional magnetic resonance imaging findings of lower activation in the frontal cortices in MA users during attention (66) and decision-making tasks (24, 25, 35, 67), reflective of reduced resources to process information and subsequent performance deficits. In addition, as MA users often lack appetite and therefore stop regular eating, nutritional effects on brain metabolism may also contribute to the observed cognitive dysfunctions (68). Our data thus further support the notion that chronic MA abuse is linked to cognitive dysfunction and may cause cognitive decline (69, 70).

Our main finding suggests an improvement of sustained attention performance when compared to levels of control subjects, while performance in controls did not improve over time. Observed effects may be specific to the treatment or represent a subgroup of patients completing treatment. The design of the present study did not include a control group for the intervention. Nevertheless, *post hoc* analysis showed that baseline performance in sustained attention was not divergent between patients who finished the program and those who prematurely stopped treatment, which allows supportive evidence for a treatment effect. This speculation however warrants further assessments. Existing cross-sectional studies already illustrate comparable neuropsychological test performance in MA users and non-MA–using controls following periods of abstinence, i.e. ≥ 8 months (16, 43, 71), reflecting that there may indeed be recovery of cognitive functioning following protracted abstinence (38). Other studies evaluating subjects with shorter periods of abstinence (5 days to 3 months) report observable deficits in a number of cognitive domains such as attention, episodic memory and executive functions (39, 41). Yet, these findings likely align with baseline deficits observed in our study with mean days of abstinence at baseline assessment in the range of these reports. Longitudinal studies that have examined the effects of abstinence on cognitive functioning in MA users when tested in early abstinence and again in later abstinence, similarly yield some evidence for functional recovery. In short observation periods (≤ 3 weeks), MA-dependent individuals have been found to improve their performance on neuropsychological tests including attention (42) and executive functioning (40). Longer periods of abstinence may also improve motor and verbal memory (36, 72). However, these studies did not include a control group for re-test effects, thus limiting conclusions on the causal role of abstinence in performance changes. The inclusion of such a control group clearly represents an important advance of the present study, strengthening our findings of attentional improvements. In support, normalization of global cognitive function in MA-dependent participants after an average of 1 year abstinence from MA has been reported in one study, which also included a control group for longitudinal effects (73).

Finally, relevant to improvements in function, there is evidence for some of the MA-associated changes on the neurobiological level to recover following periods of abstinence. This has been illustrated in human studies for MA-associated brain metabolism and monoamine system abnormalities (36, 40, 72, 74, 75) and structural alterations, e.g. prefrontal grey-matter deficit (71). Similar results have been obtained in primate (76) and rodent studies (77). Nonetheless, discordant findings in the literature examining cognitive functions and neurobiological alterations in MA users following abstinence exist [see (23)] and have been discussed to reflect differences, e.g. in study design but also important clinical characteristics such as length of abstinence. Meta-analyses, however, do not imply such an association between length of abstinence and functional impairments in MA users (58, 60).

An alternative explanation is provided if not global cognitive function but single performance domains recover over different time scales following periods of abstinence while others may even persist. This idea was put forward together with the notion that neurobiochemical alterations in the monoamine system are likely most pronounced and persistent in e.g. dopamine rich regions (72). In light of the present study this implies that performance in sustained attention, which is highly related to activity measures in the prefrontal cortices (78) and moderately innervated by dopaminergic fibers, follows an early path of recovery, while impulsive choice and decision-making are additionally dependent on high striatal activity (79), the main target of dopamine fibers. This is also in line with the idea of delay discounting representing “more” trait-like features, while sustained attention is highly state-dependent. For discounting to change, conditions must change, and the individual must adapt to the new state, which may take time but may also be drug-dependent (80).

### Clinical Relevance

Attentional ability is a critical aspect in processing environmental stimuli during decision-making and highly relevant for long-term treatment success. Pharmacological treatment studies using modafinil or ibudilast have shown some positive effects on the attentional capacity in recently detoxified MA patients (20, 81). Our data provide the first evidence that sustained attention can substantially improve during a 3-month MA-specific relapse prevention program based on cognitive behavioral therapy and motivational interviewing. This is in line with available clinical and preclinical evidence suggesting that cognitive stimulation may provide a valuable adjuvant intervention for drug addiction (82). Interestingly, a recent review shows that individual rates of delay discounting can decrease through behavioral training, endorsing context-dependent and changeable attributes in impulsive choice behavior. The most promising avenues in this regard seem to be acceptance-/mindfulness-based trainings and manipulations involving future orientation (83). Thus, we cannot exclude that impulsive choice, which did not normalize after 3 months in our study, would have improved after a longer recovery time or after implementing the aforementioned treatment modules. Although studies are required to identify explicit modules and their mechanisms, interventions that improve such cognitive domains or target activity in relevant networks are promising for the long-term reduction of MA intake and prevention of relapse.

### Limitations

It should be emphasized that this work can only be considered as a pilot study. Firstly, our findings are limited by the relatively small sample size providing low power for within-subject analyses. The small sample size additionally limited analysis to evaluate effects of medication and comorbid diagnoses. The presence of a dual diagnosis in MA users can worsen craving (84) and may thus affect behavior and relapse. While the evaluation of specific comorbid diagnoses was impossible, we could confirm our main results when including the presence/absence of psychiatric comorbidity in our model. Moreover, the number of cases with medication was too small for systematic investigations on medication effects, which represents a shortcoming as medications have been found to modulate attention performance in patients and animal models (85, 86). Secondly, no control group for intervention was included and we thus encourage similar research to address specific intervention effects. Finally, multiple measures are required to inform more rigorously about the nature and degree of deficits in different domains of attention and their developmental course under therapy. These include focused, selective, alternating and divided attention in which problems—if they significantly persist into abstinence and recovery—could result in treatment failure and return to regular MA use (87). On the other hand, our study has several strengths, exemplified by the longitudinal HC group and a naturalistic sample of MA patients with comorbid psychiatric disorders and drug abuse histories.

## Conclusion

The current study in MA patients shows that sustained attention significantly improved under treatment conditions. Our work thus lends support to the recommended psychotherapeutic interventions. Further measures of sustained attention may even present a valuable tool of parallel clinical monitoring informing treatment progress.

## Data Availability Statement

The datasets generated for this study are available on request to the corresponding author.

## Ethics Statement

The studies involving human participants were reviewed and approved by the local ethics committee of the Technische Universität Dresden. The patients/participants provided their written informed consent to participate in this study.

## Author Contributions

MP and UZ designed the study. NB, SP, and MS developed the delay discounting task. CG, AS, and MP contributed to study management, data collection and processing. RM-P, NB, JP, and SP analyzed the data. NB, JP, and MP wrote the manuscript. All authors contributed to the article and approved the submitted version.

## Funding

This work was funded by the MeDDrive program of the Technische Universität Dresden (MeDDrive grant #60.401) and the Deutsche Forschungsgemeinschaft (DFG, German Research Foundation) – Project-ID 402170461 – TRR 265 (88). The funding bodies had no further role in the conceptualization of the study, the collection and analysis of data, the preparation of the manuscript or the decision to publish.

## Conflict of Interest

The authors declare that the research was conducted in the absence of any commercial or financial relationships that could be construed as a potential conflict of interest.

The handling editor declared a shared affiliation, though no other collaboration, with the authors at the time of review.
