# Supplementary material for: Neurocognitive Dysfunctions and Their Therapeutic Modulation in Patients With Methamphetamine Dependence: A Pilot Study
Source: Front Psychiatry. 2020 Jul 2;11:581. doi: 10.3389/fpsyt.2020.00581 (PMC7343893; doi:10.3389/fpsyt.2020.00581)
Supplement: Supplementary file 1 [file Image_1.pdf]

Neurocognitive Dysfunctions and Their Therapeutic Modulation in Patients With Methamphetamine Dependence: A Pilot Study

Nadine Bernhardt[1*†], Johannes Petzold[1†], Cornelius Groß[1], Anna Scheck[1], Shakoor Pooseh[2], René Mayer-Pelinski[1], Ulrich S. Zimmermann[1,3], Michael N. Smolka[1], Maximilian Pilhatsch[1,4†]

[1]Department of Psychiatry and Psychotherapy, Technische Universität Dresden, Dresden, Germany

[2]Freiburg Center for Data Analysis and Modeling, Albert-Ludwigs-Universität Freiburg, Freiburg, Germany

[3]Department of Addiction Medicine and Psychotherapy, Isar-Amper-Klinikum München-Ost, Haar, Germany

[4]Department of Psychiatry and Psychotherapy, Elblandklinikum Radebeul, Radebeul, Germany

***Correspondence:**

Nadine Bernhardt, nadine.bernhardt@uniklinikum-dresden.de

†ORCID:

Nadine Bernhardt, orcid.org/0000-0002-3188-8431

Johannes Petzold, orcid.org/0000-0003-4163-9014

Maximilian Pilhatsch, orcid.org/0000-0003-4323-3309

Keywords: Methamphetamine, Crystal Meth, Sustained Attention, Delay Discounting, Psychotherapy

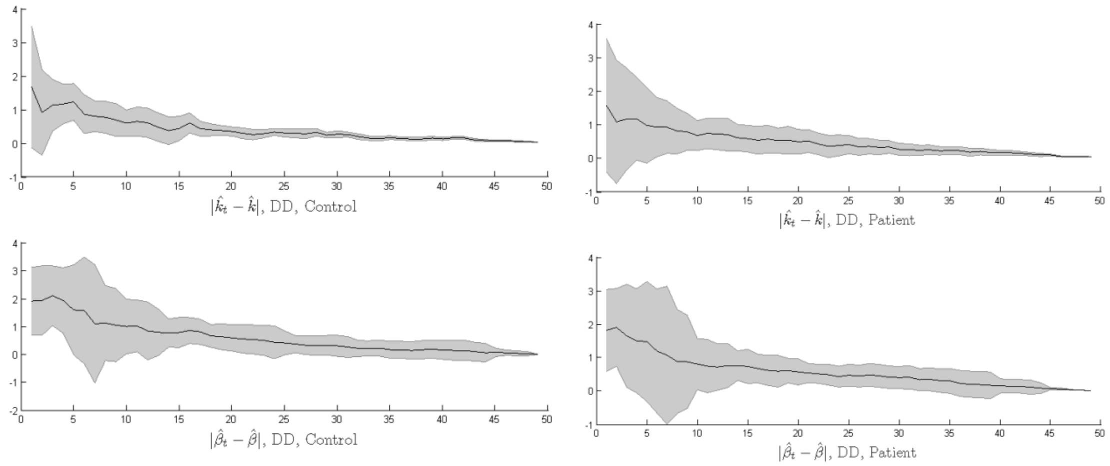

Supplementary Figure 1. Convergence of parameter estimation in sample data of control subjects and methamphetamine patients. The average absolute differences between the estimation at each trial and the final estimation for all participants in the respective group are shown trial by trial by black lines. The gray area depicts the standard deviation distance from the average. The decreasing pattern in black lines is a sign of convergence and the same for standard deviations, meaning that this is true for the whole group. The top row depicts the

estimation of the behavioral delay discounting parameter *k*, the bottom row the consistency of choice using parameter *β*.

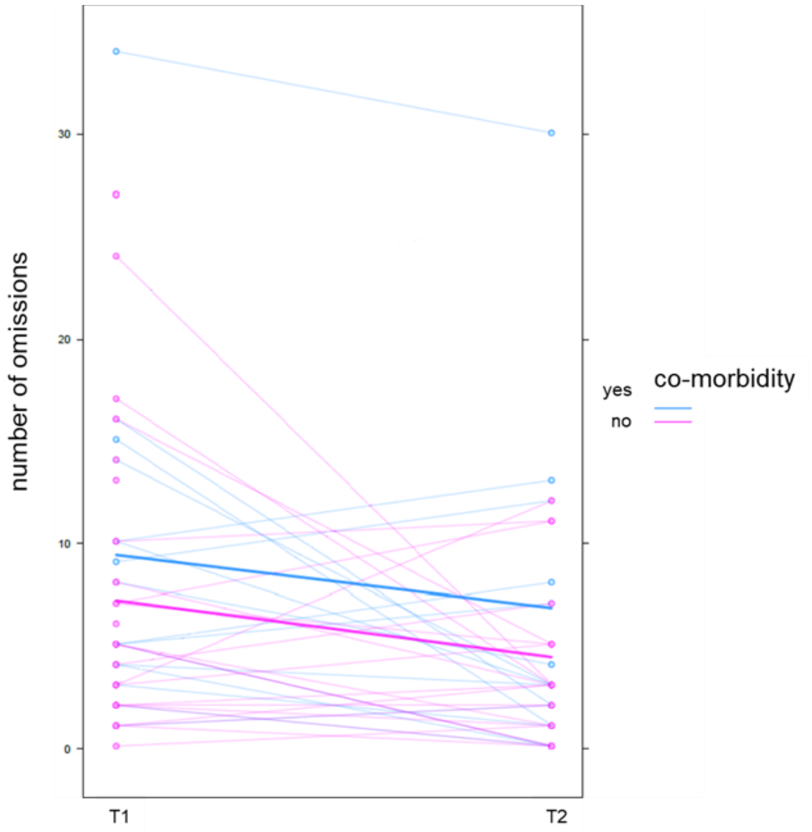

Supplementary Figure 2. Sustained attention showed decline when controlled for co-morbidity in patients (yes, no). Analysis of omissions over time points in methamphetamine patients showed a significant effect of time (Estimate = -4.35, SD = 1.71, t = -2.536, p = .0019) but no effect of co-morbidity (Estimate = 2.49, SD = 2.79, t = 0.892, p = .3797). Linear mixed model fit by REML t-tests use Satterthwaite approximations to degrees of freedom [lmerMod]. Group means are in bold.

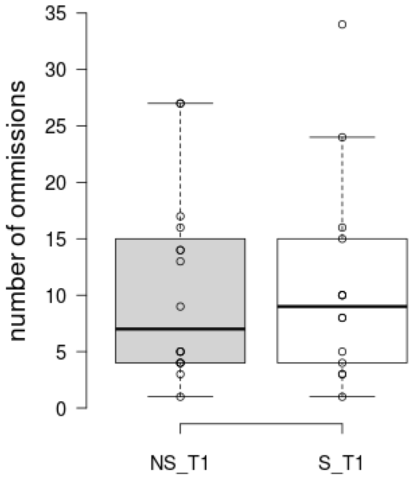

Supplementary Figure 3. Comparison between baseline performance of patients with unsuccessful (NS_T1) and those with successful treatment outcome (S_T1) illustrated that sustained attention was no prerequisite for compliance but associated with treatment, T(28) = 0.090, p = .929.
